# '*In situ* simulation' versus 'off site simulation' in obstetric emergencies and their effect on knowledge, safety attitudes, team performance, stress, and motivation: study protocol for a randomized controlled trial

**DOI:** 10.1186/1745-6215-14-220

**Published:** 2013-07-17

**Authors:** Jette Led Sørensen, Cees Van der Vleuten, Jane Lindschou, Christian Gluud, Doris Østergaard, Vicki LeBlanc, Marianne Johansen, Kim Ekelund, Charlotte Krebs Albrechtsen, Berit Woetman Pedersen, Hanne Kjærgaard, Pia Weikop, Bent Ottesen

**Affiliations:** 1 Department of Obstetrics, Department of Anaesthesia and Juliane Marie Centre for Children, Women and Reproduction, Rigshospitalet, Copenhagen University Hospital, Blegdamsvej 9, Copenhagen, Denmark; 2 Department of Educational Development and Research, Faculty of Health, Medicine and Life Sciences, Maastricht University, P.O. Box 616, 6200, MD Maastricht, The Netherlands; 3 Copenhagen Trial Unit, Centre for Clinical Intervention Research, Rigshospitalet, Copenhagen University Hospital, Blegdamsvej 9, Copenhagen, Denmark; 4 Danish Institute for Medical Simulation, Herlev Hospital, Capital Region of Denmark and Copenhagen University, Herlev Ringvej 75, 2730 Herlev, Denmark; 5 The Wilson Centre, University of Toronto, 200 Elizabeth Street, 1ES-565, Toronto, Ontario M5G 2C4 Canada; 6 Laboratory of Neuropsychiatry, Rigshospitalet, Copenhagen University Hospital, Blegdamsvej 9, Copenhagen, Denmark

**Keywords:** Simulation, *In situ* simulation, Randomized trial, Obstetric emergencies, Multi-professional education, Stress

## Abstract

**Background:**

Unexpected obstetric emergencies threaten the safety of pregnant women. As emergencies are rare, they are difficult to learn. Therefore, simulation-based medical education (SBME) seems relevant. In non-systematic reviews on SBME, medical simulation has been suggested to be associated with improved learner outcomes. However, many questions on how SBME can be optimized remain unanswered. One unresolved issue is how '*in situ* simulation' (ISS) versus 'off site simulation' (OSS) impact learning. ISS means simulation-based training in the actual patient care unit (in other words, the labor room and operating room). OSS means training in facilities away from the actual patient care unit, either at a simulation centre or in hospital rooms that have been set up for this purpose.

**Methods and design:**

The objective of this randomized trial is to study the effect of ISS versus OSS on individual learning outcome, safety attitude, motivation, stress, and team performance amongst multi-professional obstetric-anesthesia teams.

The trial is a single-centre randomized superiority trial including 100 participants. The inclusion criteria were health-care professionals employed at the department of obstetrics or anesthesia at Rigshospitalet, Copenhagen, who were working on shifts and gave written informed consent. Exclusion criteria were managers with staff responsibilities, and staff who were actively taking part in preparation of the trial. The same obstetric multi-professional training was conducted in the two simulation settings. The experimental group was exposed to training in the ISS setting, and the control group in the OSS setting. The primary outcome is the individual score on a knowledge test. Exploratory outcomes are individual scores on a safety attitudes questionnaire, a stress inventory, salivary cortisol levels, an intrinsic motivation inventory, results from a questionnaire evaluating perceptions of the simulation and suggested changes needed in the organization, a team-based score on video-assessed team performance and on selected clinical performance.

**Discussion:**

The perspective is to provide new knowledge on contextual effects of different simulation settings.

**Trial registration:**

ClincialTrials.gov NCT01792674.

## Background

Care for pregnant and parturient women is a field where unexpected emergencies occur; for example, emergency Caesarean section, postpartum bleeding or severe pre-eclampsia, that may potentially harm both mother and baby [1-4]. Since obstetric emergencies are rare and hence by nature difficult to learn in real life, simulation-based medical education (SBME) is argued to be an essential remedy [5]. SBME is defined as “a person, device, or set of conditions which attempts to present education and evaluation problems authentically. The student or trainee is required to respond to the problems as he or she would under natural circumstances” [6].

Labor wards have a dual function in creating a relaxed atmosphere for normal childbirth and at the same time showing readiness to deal with life-threatening emergencies [7]. Labor wards are challenging work places and patient safety and medical litigation are high on the agenda [8-11]. In certain situations, clinical management of pregnant and parturient women may require the involvement of a variety of health-care professionals and medical specialties. The primary care team in a delivery room consists of a midwife assisted by an auxiliary nurse. In cases of emergencies, more experienced midwives and obstetricians will be called for assistance. If the clinical situation progresses further to an emergency, an anesthesiologist, a nurse anesthetist and the operating room personnel may become involved. Occasionally, involvement of other specialties may also be required, when a rather common clinical event has evolved into a potentially life-threatening situation calling for multi-professional and multi-disciplinary clinical management.

Such rare and complex clinical situations require complex skills, which cannot be trained and learned in clinical practice. Thus, there is a need for SBME in obstetric emergencies. In a systematic review of training in acute obstetric emergencies [12] the authors applied the quality assessment of diagnostic accuracy studies criteria. Out of 97 articles, only eight articles - four randomized trials and four cohort studies - assessing the effect of teamwork training in a simulation setting were identified. Based on these trials, it was concluded that teamwork training in a simulation-based setting resulted in improvements in knowledge, practical skills, communication, and team performance in acute obstetric situations. No difference in outcomes was found when comparing SBME in a dedicated simulation centre with SBME in a local hospital setting [13,14].

From the non-systematic reviews on SBME [6,15] and the obstetric systematic review [12] we can conclude that SBME in labor wards is worthwhile, and that multi-professional and multi-disciplinary team training are important approaches due to the complexities of the trained skills and the rarity of the high-risk obstetric emergencies. However, we need to further study key elements of SBME in order to fully understand how we can best improve SBME in obstetric emergencies. One potential element influencing the effect of simulation might be the level of authenticity of the simulation or, in other words, the fidelity of the simulation. Fidelity is described as a multi-dimensional concept consisting of different parts: 1) physical/functional or engineering fidelity, which mean the degree to which the simulator duplicates the appearance and perception of the real system; 2) psychological fidelity is the degree to which the trainee perceives the simulation to be an authentic surrogate for the trained task. The literature states that psychological fidelity is considered to be the most essential requirement when conducting team training [16,17]. The simulation setting has traditionally been 'off site simulation' (OSS), either at a simulation centre or in local facilities in the hospital set up for the single purpose of simulation training. However, more recently, a new simulation modality, the '*in situ* simulation' (ISS), has been introduced. ISS is described by Riley and colleagues [18] as “a team-based simulation strategy that occurs on the actual patient care units involving actual healthcare team members within their own working environment”. An unanswered question is whether ISS is superior compared with OSS with regards to simulation-based learning in obstetric emergencies? We hypothesized that the psychological fidelity is influenced by the setting in which the simulation training is conducted, and that ISS can add to the level of fidelity and therefore be more effective.

Apart from a few larger observational studies within different medical specialties [18-20], most of the studies conducted on ISS describe a local educational intervention with a local ISS program. Methodologically, the studies are descriptive and few include a control group or pre- and post-tests, and we have not been able to identify any randomized trials [21]. It is argued that ISS can identify system weaknesses because ISS takes place in the real working environment and, therefore, potentially has more psychological fidelity as opposed to OSS [18-22], and ISS can be used to test how new processes are functioning in clinical facilities [23]. Some argue that ISS overcomes feasibility issues and is cost saving compared to OSS in simulation centers [24,25]. ISS can consist of either an announced training event or an unannounced event. Anderson and colleagues [26] focused on unannounced ISS and its potential disadvantages, and argued how unannounced ISS is time consuming and may intimidate participants.

Human factors such as stress and motivation impact learning [27-31]. Studies show that simulation can be a stressor. High stress responsiveness has been associated with both enhanced and impaired performance, but with enhanced learning [29]. As such, further exploration of these issues is needed. Experimental studies have used unspecific measurements of stress level [32], and different stress inventories as well as measurements of salivary cortisol levels [28,33-36]. Motivational processes are central to learning [27,30,37], and as part of this trial we will investigate phenomena such as intrinsic motivation, and how this is moderated by the two different training settings (ISS versus OSS). We hypothesize that, in simulation-based training in obstetric emergencies, ISS is more effective than OSS regarding learning. We anticipate that the participants will experience ISS as more demanding, and that ISS will create higher levels of stress and motivation, which may yet again enhance learning. Further, we hypothesize that ISS training may provide the investigators with more information on changes needed in the organization than OSS training will. Randomized trials are needed to obtain knowledge on the effect of ISS versus OSS on participants and its advantages and disadvantages.

## Methods and design

The design is a single-center, investigator-initiated randomized superiority trial.

### The setting

The trial was undertaken at Rigshospitalet, Copenhagen University Hospital, in an obstetric and anesthesia high-risk department with approximately 6,600 deliveries per year. The intervention period was scheduled to run through April to June 2013, and follow-up by questionnaires until August 2013.

### Participants

All health-care professionals from the department of obstetrics and anesthesia, Juliane Marie Centre for Children, Women and Reproduction, Rigshospitalet, working on or in relation to the labor ward, were eligible for inclusion in the trial. These health-care professional groups, who were working on shift, encompassed: specialized obstetricians; trainee obstetricians; midwives; specialized midwives; auxiliary nurses; specialized anesthesiologists; trainee anesthesiologists; nurse anesthetists; and surgical nurses. Participants gave informed consent. Exclusion criteria were lack of informed consent, employees with managerial and staff responsibilities, staff members involved in the design or conduction of the trial, and finally employees who did not work in shifts.

### Randomization

Randomization was done by the Copenhagen Trial Unit, using a computer-generated allocation sequence concealed to the investigators. The randomization was conducted in two steps. The participants were individually randomized into the experimental group (ISS) or the control group (OSS). The allocation sequence was stratified according to health-care professional groups in order to resemble authentic teams and according to the days they were available for training. After individual randomization, the participants in either group (ISS and OSS) were randomized into five teams each.

### Trial interventions

This trial included an experimental educational intervention ISS [18,21], which means training in the actual patient care unit (in other words, the labor room and operating theatre). The experimental intervention in the present trial was pre-announced ISS. We planned to conduct announced ISS training, as the complexity of conducting unannounced ISS sessions with the involvement of health-care professionals on a larger scale is unrealistic taking into consideration work schedules and the daily clinical work activities. Training of the control group (OSS) took place in training rooms that were set up for the occasion in the hospital, but away from the actual patient care unit.

The simulated scenarios applied in the trial were contained in a full training day. The development of the curriculum for the training day was based on an instructional design approach [38,39] and was developed and pilot tested by a local multi-professional working committee. In January 2012 this working committee was appointed by the managerial groups of the departments of anesthesia and obstetrics and consisted of representatives from all the health-care professionals who will participate in the trial. This working committee developed aims and objectives based on the principles of Blooms taxonomy [40], and the aims and objectives were approved by the management groups. The simulated scenarios in ISS and OSS were designed in a way that involved both the labor room setting and the operating theatre, to specifically focus on the patient journey and the communication amongst health-care professionals during patient transfers, where many different health-care professionals and different medical disciplines are involved. This approach to training was chosen based upon previous experience with obstetric simulation-based training conducted in the obstetric department [32,41] and was designed in accordance with the overall plan of strategy of the obstetric department and the anesthesia department, Juliane Marie Centre for Children, Women and Reproduction, Rigshospitalet [42].

In the labor room, a simulated patient acted as the patient. In the operating room, a full body interactive birthing simulator, a SimMom, was the patient [43]. The SimMom simulator offers the functionality required for training in a wide range of midwifery, obstetric and anesthesia skills, and the anatomy and functionality of the SimMom allows for multi-professional training of labor and delivery management. Standardized clinical simulated scenarios were designed and, combined with pre-programmed scenarios on the SimMom, this allowed for standardized training. The educators were recruited from the local working committee, and all educators were trained to run the scenarios in a standardized way and in facilitating the simulation scenarios and debriefing the participants.

**Figure 1:**
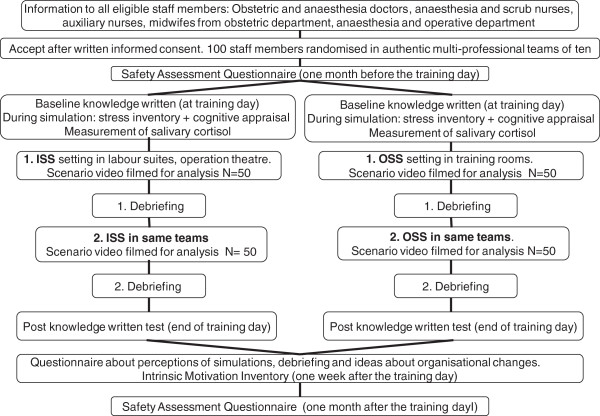
**Randomized trial of ‘*in situ *simulation’ (ISS) versus ‘off site simulation’ (OSS): randomization, intervention and outcome measurements.**

In addition, the training day also included some video-based, case-based and lecture-based teaching sessions. Also, on the simulation days, data related to the trial were collected in the form of written multiple choice questions (MCQ), questionnaires on subjective stress and salivary cortisol samples (Table 1). Training days were scheduled into the individual employee’s working plan. Figure 1 gives an overview of the randomization and intervention procedure as well as outcomes.

**Table 1:** Time schedule of measurements

**Individual measurements**							**Team measurements**	
**Multiple choice questionnaire**	**Safety attitudes questionnaire**	**Stress**-**trait anxiety inventory**	**Cognitive appraisal**	**Test for salivary cortisol**	**Intrinsic motivation inventory**	**Evaluation questionnaire**	**Team emergency assessment measure**	**Selected clinical measures**
Training day start of day	4 weeks before training day	Training day before 1. simulation and twice after	Training day before 1. simulation and twice after	Training day before 1. simulation and three times after	1 week after training day	1 week after training day	Training day	Training day
							1. simulation: video recordings. Video assessment by independent assessors.	1. simulation: video recordings. Video assessment by independent assessors.
Training day end of the day	4 weeks after training day	Training day before 2. simulation and twice after	Training day before 2. simulation and twice after	Training day before 2. simulation and three times after			Training day	Training day
							2. simulation: video recordings. Video assessment by independent assessors	2. simulation: video recordings. Video assessment by independent assessors

### Blinding

The participants and the educators providing the educational intervention, and the assessors observing and assessing videos, were not blinded to the intervention. The data managers, statisticians and investigators drawing conclusions will be blinded to the allocated intervention groups.

### Measurements and assessment of outcomes

Table 2 provides an overview of the variables, outcomes and accompanying statistical analyses. Table 2 is inspired by the SPIRIT 2013: Explanation and elaboration: guidance for protocols of clinical trials [44]. See Table 1 for the time schedule for obtaining measurements.

**Table 2:** **Variables**, **research hypothesis**, **outcome measures and methods of statistical analysis**

**Variable/outcome on individual level (N = 100)**	**Research hypothesis**: **experimental group versus control group**	**Outcome measure**	**Type of variable**	**Methods of statistical analysis**
**Primary outcome**				
Multiple choice questions	Improvement occurs in the experimental group	Percentage correct in 40 multiple choice questions	Will be analyzed as interval data, a Gaussian distribution is expected	Parametric techniques
				ANOVA
**Exploratory outcome**				
Safety Attitudes Questionnaire	Increased score in the experimental group	33 items on a 5-point scale. Divided into 6 dimensions	Will be analyzed as interval data, a Gaussian distribution is expected	Parametric techniques
				ANOVA
		Data are converted to the 100-point scale		Chi-square tests
Stress-Trait Anxiety Inventory Baseline	Increased peak score in the experimental group	Inventory 20 item (interval 20 to 80).	Will be analyzed as interval data, a Gaussian distribution is expected	Parametric techniques
				ANOVA
Stress-Trait Anxiety Inventory 1				
Stress-Trait Anxiety Inventory 2				
Cognitive appraisal A Baseline	Increased peak score in the experimental group	Likert scale 1 (10 point)/Likert scale 2 (10 point) (interval 1/10 to 10)	Ordinal data	Non-parametric techniques
Cognitive appraisal 1				Mann Whitney U test
Cognitive appraisal 2				
Test for salivary cortisol Baseline	Increased salivary cortisol level from baseline to peak in the experimental group	Cortisol level in nmol/l	Interval data	Parametric techniques
Test for salivary cortisol 1				ANOVA
Test for salivary cortisol 2				
Test for salivary cortisol 3				
Evaluation questionnaire	Increased positive evaluation in the experimental group	20 questions on a 5-point Likert scale	Ordinal data	Non-parametric techniques
				Treated as ordinal data at the item level
Intrinsic Motivation Inventory	Increased score in the experimental group	Task evaluation inventory 22 items on a 7-point scale. Divided into 4 dimensions	Ordinal data	Non-parametric techniques
				Mann Whitney U test
**Variables on team**-**level**				
**(N = 10 teams)**				
Team Emergency Assessment Measure	Improved outcome in the experimental group	Video assessment on a 5-point scale (0 to 5) of 11 questions (0 to 44) 10 points scale for global rating of the team	Ordinal data	Non-parametric techniques
				Mann Whitney U test
Selected clinical measures	Improved outcome in the experimental group	Minutes before decision making about operation, minutes before operation initiated. Medication given yes/no	Interval data	Parametric techniques

The table is inspired by SPIRIT 2013: Explanation and elaboration: guidance for protocols of clinical trials [44]. ANOVA, analysis of variance.

### Primary outcome

The primary outcome is knowledge test results from MCQs. The mean values of results of the MCQs of the experimental and control group were tested at the end of the training day and will be compared.

Previous research on knowledge testing has found that written tests are able to predict results in performance-based testing [45,46]. The argument for applying the MCQ is that it is feasible to test many participants in a relatively short time and at low costs [45]. Previously used MCQ tests and 'knowledge of skills test' [13,32] were used for inspiration, when constructing this new MCQ.

The MCQs were created as a 'one-best-answer' item format with three to five options, which requires the participants to select the single best response [47,48]. The content of the MCQs were based on aims and objectives developed by the multi-professional working group appointed by the management and has been tested amongst all health-care professional in this local working group. The content validity was further tested among specialized obstetricians and specialized obstetric anesthesiologists. Subsequently, the MCQs were tested among midwifery students, medical students, trainee doctors and specialized obstetricians and specialized obstetric anesthesiologists from other hospitals and were found to be construct valid. During the statistical analysis, some items in the MCQ needed to be deleted. The description of development and results of the MCQs used in this trial will be reported in another publication.

### Exploratory outcomes

The “Safety Attitudes Questionnaire” (SAQ) consists of 33 items on a five-point scale that is divided into six dimensions. SAQ was applied approximately 1 month prior to and approximately 1 month after the training day. The mean values on six different dimensions of the SAQ results from the experimental and control group will be compared 1 month after the training day. SAQ is an inventory used in several countries and also applied and validated in a Scandinavian context, and previously tested in Denmark [49-51].

Salivary cortisol (reflecting the hypothalamic-pituitary-adrenal axis activity) was used as a biological marker of stress levels. The Sarstedt Cortisol Salivette Device, provided by Neogen corporation 944 Nandino Blvd, Lexint KY 40511–1205 USA Product no. 402710, was used The analysis will be a duplicate analysis based on the Elisa Technique 405 nm, where 100 ul sample will be extracted and 50 ul in duplication will be used in the ELISA kit. Eight standards in ranges from 0.04 ng/ml up to 10ng/ml will be used in the assay together with a blanc control. The microtitterplate will be read in a dual wavelength set at 450 nm and 650 nm. In calculation of the data, the blanc background will be subtracted from all absorbance values before a non linear fit to the standard curve will be calculated.

The salivary cortisol sample was obtained before the simulation (baseline) and three times in relation to the simulations. The cortisol response will be measured as increased salivary cortisol from individual baseline to peak, and mean response values in the experimental and control group will be compared.

State-Trait Anxiety Inventory (STAI-1) was administered before the simulations started (baseline) and twice following the simulations [52,53]. It will reflect the subjective stress response. The peak level of subjective stress response will be used and mean values in the experimental and control group will be compared.

Cognitive appraisal [31,36,54] was assessed before and after each scenario, using the method described by Tomaka [54]; in other words, primary appraisal was examined by asking the participants to answer the question “how stressful do you expect the upcoming task to be?” Secondary appraisal was measured by asking the participants “how able were you to cope with this task?” The participants indicated their answers on an anchored ten-point Likert scale. An index of cognitive appraisal will be calculated as the ratio of the primary appraisal (task) to the secondary appraisal (resource). If the resources are assessed as being greater than the task demands, the situation is appraised as a 'challenge'. If the task demands were appraised as being greater than the resources, the situation is appraised as a 'threat' [54].

“Intrinsic Motivation Inventory” consists of 22 items on a seven-point scale that is divided into four dimensions. It was administered as a questionnaire approximately 1 week after the training day [27]. The median values in the experimental and control group will be compared.

A questionnaire was administered to evaluate participant perceptions of the simulations and the debriefing approximately 1 week after the training day. This questionnaire included questions on a Likert scale about personal perceptions of the scenario (that is, learning, realism, cooperation between health-care professionals, own role in the team, et cetera) and whether the simulation training scenarios inspired the participants to suggest organizational change proposals (that is, changes in guidelines, practical things, et cetera). The data will be treated as ordinal data at the item level. The median values in the experimental and control group will be compared.

Team performance score will be assessed by independent observers through reviewing video recordings of the scenarios. A validated rating scale “Team Emergency Assessment Measure” developed by Cooper and colleagues [55,56] will be used. The median scores of the performance in the experimental and control group will be compared.

Clinical performance in the simulated setting will be assessed by the independent assessors through the reviewing of video recordings of the scenarios. The assessment score is based on data such as minutes passed from the scenario starts till decision was made about operation, minutes from decision making before operation was initiated, and whether medications such as uterotonics were administered or not. The mean score of the performance in the experimental and control group will be compared.

### Sample size calculation

There are no data on training effectiveness of ISS upon which to base sample size calculations. We chose to calculate the required sample size based on experience with knowledge tests from data in previous studies [13,32]. We were planning a trial of a continuous response variable from independent control and experimental participants with one control per experimental participant. We assumed the response within the experimental and the control group to be normally distributed with a standard deviation of 24%. If the true difference in the experimental and control means was 17%, we needed to study 32 experimental participants and 32 control participants (a total of 64) to be able to reject the null hypothesis; that is, that there was no difference in population means of the experimental and control groups with a probability of (power) 80%. The two-sided type I error probability associated to test this null hypothesis was 5%.

### Sample size estimation adjusted for clustering

As the intervention was delivered in teams (clusters), observations on participants in the same team were likely to be correlated. Hence the effective sample size was less than that suggested by the actual number of individual participants. The reduction in effective sample size depends on the intra-class or cluster correlation coefficient (ICC) [57,58]. In order to adjust the sample size for this, the crude sample size calculated above needed to be multiplied by the design effect. The cluster size was ten, as there were ten participants in each team, and we assumed the ICC to be 0.05 [58]. Design effect = 1 + (cluster size – 1) × ICC → design effect = 1.45. Accordingly, the sample size was then 64 × 1.45 = 92.8 participants. We therefore planned to include 100 participants in the experimental and control groups (50 in each group) each of which consists of five teams of 10 participants in each arm. Statistical methods for the primary and exploratory outcome of the hypotheses are laid out in Table 2, and also the statistical methods are described. The intervention was delivered in teams, which means that participants were clustered within teams. Since observations from individuals in the same team are potentially correlated we will use generalized estimating equations (GEE) [59] in the parametric analyses to take this cluster effect into account. The statistical analysis will be adjusted for health-care professional groups. The experimental group (participants or teams in ISS) will be compared against the control group (participants or teams in OSS) for all analyses. The results will be expressed by means with standard deviations and confidence intervals, as well as by medians with percentiles. Associated *P*-values and effect sizes will be reported.

For the interval scale data, linear regression will be used to analyze changes between the experimental and the control group from baseline to peak. GEE will be used to take the clustered nature of the data into account. Non-parametric statistical analyses will be used for the ordinal scale data. Medians and percentiles will be reported and the Mann–Whitney U test will be used. Individual responses to the evaluation questionnaire are measured on a Likert scale and will be treated as ordinal data and analyzed at the item level.

To take missing data into account, all analyses will be performed as intention-to-treat analyses. Missing data will be handled by multiple imputation techniques. For all tests, we will use 2-sided *P*-values with alpha < 0.05 being the level of significance. We will use the Benjamin-Hochberg method to adjust for multiple testing [60].

### Ethical consideration

Participants are health-care professionals and neither patients nor patient data are used in the trial. The trial complies with the current version of the Declaration of Helsinki on biomedical research and with the Act on Processing of Personal Data. Relevant approval from The Regional Ethics Committee (protocol number H-2-2012-155) and the Danish Data Protection Agency (Number 2007-58-0015) are obtained. The trial is registered at http://www.clinicaltrials.gov with number NCT01792674.

The training program was planned to take place during normal working hours and participants were paid full salary for their attendance. No further compensation was given to participants. Participation was voluntary and the participants could withdraw from the trial at any time.

Participants were assured that their personal data, data on questionnaires, salivary cortisol samples and video-recordings will remain anonymous during analyses and reporting. The participants were asked to respect the confidentiality of their observations about colleagues’ performance in the simulated setting.

### Recruitment of participants

The eligible participants were informed at conferences, meetings, on a web page [61], by written notice on notice boards, and by a personal letter administered by the hospital local post distribution, which gave the participants the opportunity to make an informed decision about their participation in the trial. The eligible participants could obtain more written information from our web page [61] and by contacting the principal investigator or another contact person directly. After receiving written and verbal information, eligible participants were asked to sign a consent form before being enrolled in the trial.

## Discussion

This is the first randomized trial investigating the effect of ISS versus OSS for SBME. An advantage of the trial is that it includes authentic teams of health-care professionals also involved in these clinical scenarios in real life. Several simulation-based studies are not performed on authentic teams and students have often been enrolled as they are more flexible and easier to include in trials. However, applicability of these data is questionable as results based upon undergraduate students may not necessarily apply to postgraduate employed health-care workers. Including authentic teams will probably be advantageous when interpreting the results and drawing conclusions.

However, the fact that authentic obstetric-anesthesia teams are trial participants - that is, fully employed health-care professionals - may carry feasibility problems. There will be a risk that situations arise in which real emergencies combined with lack of staff necessitate that some of the randomized health-care professionals will need to discontinue the trial participation. Further, there is a minor risk that a full team randomized to ISS needs to discontinue if a real life emergency situation necessitates the use of the rooms in the labor ward and operating theatre that were allocated to the trial for the day. Through our careful planning and cooperation with the managerial teams of the involved departments, this risk will be minimized.

A potential weakness is the fact that the trial is a single site trial, including only a moderate number of participants. There will also be a risk of contamination amongst teams, as the health-care professionals in the experimental team (ISS) intermingle with staff members allocated to the control group (OSS) and may share information. This may affect the generalizability of this study. Moreover, this trial only assesses surrogate outcomes for the relevant clinical outcome, that is, whether neonatal and maternal health fares better with ISS compared with OSS. However, being the first randomized trial comparing ISS with OSS, the trial has the potential to add some new insight with regards to the effect of authenticity in the setting for SBME and to inform future research in this field.

The sample size estimation has been based on data from other knowledge tests [13,32], as there are no current data on knowledge testing, and on the effect of training effectiveness of ISS versus OSS. The sample size calculation is adjusted for clustering. However, we have no prior information about the ICC, and therefore this estimation is based on general recommendations [57,58].

The primary outcome is a knowledge test. As alluded to above, it would have been more optimal to have neonatal and maternal health as clinical outcomes. However, this is not possible in the present trial, as a very high number of deliveries will be required to directly measure patient-relevant outcomes in obstetrics [62]. However, there are educational studies indicating that a performance in a written knowledge test can relate to clinical performance in practice [63].

Given the nature of the trial, it will not be possible to blind the participants, the educators providing the educational intervention, or the assessors observing and assessing videos. This will give a risk of overestimating the beneficial effects of the experimental intervention [64,65]. However, the allocated intervention group will be blinded for the data managers, statisticians and investigators drawing conclusions, and we will consider the risks of bias when drawing conclusions.

This trial can bring new information on SBME. The simulation setting has traditionally been OSS; however, an unanswered question is which advantages, if any, ISS can add to learning. Randomized trials are needed to obtain knowledge of advantages and disadvantages of ISS versus OSS. The study can potentially also inform the theory of fidelity of simulation [16]. The results of this trial may also add knowledge to inform the political planning and decision making process during rebuilding and building of hospitals and simulation centers. It is important to know whether high-fidelity simulation centers should be prioritized as opposed to designing/building simulation rooms 'in situ' for future simulation-based education.

## Trial status

Planning of the trial was initiated in January 2012. Enrolment of participants was initiated in January 2013. The intervention is scheduled to start in April 2013 and will stop in June 2013. Follow-up by questionnaires will continue until August 2013.

## Abbreviations

GEE: Generalized estimating equations; ICC: Intra-class or cluster correlation coefficient; ISS: *In situ* simulation; MCQ: Multiple choice questions; OSS: Off site simulation; SAQ: Safety Attitudes Questionnaire; SBME: Simulation-based medical education.

## Competing interests

The authors declare they have no financial or academic competing interests.

## Authors’ contributions

JLS created the idea of the trial. All authors made contributions to the design of this trial. BO and CVdV are supervisors of the trial. Acquisition of funding was done by JLS, supported by BO and HK. JLS, JL and CG have contributed to the sample size estimation and detailed designing of and execution of the randomization process. JLS, MJ, CKA, KE, BWP, DØ and VL made substantial contributions to the practical issues and logistics of the trial. PW contributed to the discussion, practical issues and logistics about testing salivary cortisol. JLS wrote the draft manuscript. All authors provided critical review of this paper and approved the final manuscript.
